# Inside the kaleidoscope: unravelling the “feeling different” experience of bicultural bilinguals

**DOI:** 10.3389/fpsyg.2024.1376076

**Published:** 2024-05-21

**Authors:** Silvia Purpuri, Claudio Mulatti, Roberto Filippi, Barbara Treccani

**Affiliations:** ^1^Department of Psychology and Cognitive Sciences, University of Trento, Trento, Italy; ^2^IOE, UCL’s Faculty of Education and Society, London, United Kingdom

**Keywords:** language, culture, bicultural bilinguals, migration, feeling different

## Abstract

This article explores the phenomenon of “feeling different” experienced by bicultural bilingual individuals when they switch between their two different languages. Available data suggests that this experience is genuine and holds substantive value, not merely anecdotal. While on one hand, such a feeling may stem from the fact that the two languages were acquired at different times in individuals’ lives (with all that entails in terms of efficiency and empowerment in using the two languages), on the other hand, it seems to entail deeper differences linked to the differential activation of cultural values, behavioral patterns, and expectations when the two languages are used. Its manifestations seem to be influenced by a variety of factors beyond just language choice, including the context in which this choice is performed. Results of studies investigating the experience of feeling different also suggest that it can lead to a sense of exclusion, isolation or marginalization within one’s own community. However, this experience more often yields positive outcomes, with individuals perceiving it as enriching and contributing positively to both their personal development and the broader societal fabric. Amid contemporary challenges related to immigration, the study of biculturalism and related psychological phenomena, such as the “feeling different” experience, becomes imperative, as it may provide insights into how individuals navigate the complexities linked to their cultural identities.

## Introduction

In a progressively globalized world, the concept of cultural diversity has gained tremendous significance. As globalization continues to bridge borders and bring people together, and immigration becomes more frequent, the prevalence of bicultural bilingual individuals is increasingly evident. Bicultural bilinguals, who internalize diverse cultural frameworks and languages, represent a vibrant and expanding segment of our societies. However, beneath the surface lies an underexplored phenomenon: the feeling of being different experienced by these individuals when navigating their dual cultural and linguistic identities.

### Biculturalism

Biculturalism refers to the coexistence of two distinct cultures within an individual, group, or society (Berry, 1997). It has been defined in many ways. A bicultural person, according to Grosjean (2008), is characterized by at least three traits. They participate in the life of two cultures, they adapt to them, and they combine and blend aspects of both cultures involved. The term biculturalism is used to describe the integration of elements from two separate cultural backgrounds, allowing individuals to navigate and adapt to both cultural environments effectively (LaFromboise et al., 1993). People become bicultural because, at some point in their lives, they come in contact with other cultures and live, to various degrees, with them (Grosjean, 2015). This often occurs when individuals are exposed to or grow up in two different cultural settings, such as, for instance, those people who have parents from different cultural backgrounds, or those who have migrated to a new country and have adopted the new culture while maintaining their original cultural identity (Phinney and Devich-Navarro, 1997). Biculturalism entails proficiency in two cultures, reflected in language use, friend choice, and media preferences (Cabassa, 2003). True biculturalism involves integrating cultures into a personalized blend, creating an individualized ‘idioculture’ (Benet-Martínez et al., 2002; Nguyen and Benet-Martinez, 2010).

### Bilingualism

Bilingualism, historically seen as mastering two languages equally (Bloofield, 1935), is now recognized as involving varied language use for different purposes and contexts, with differing proficiencies (Grosjean, 2010, 2013; Wei, 2020). Bilingualism has to be considered as something relative (Mackey, 2000). It is extremely difficult, if not even impossible to define precisely who is or is not bilingual (Baker, 2006).

Language ability is typically measured in two productive parts, speaking and writing, and two receptive parts, listening and reading. While some are balanced bilinguals, most use their languages in varying proportions worldwide. Authors have categorized bilinguals based on the timing of their second language acquisition (Birdsong, 1992; Genesee et al., 1995; Flege et al., 1999). However, distinguishing types can be challenging for casual observers, as all may achieve full proficiency.

### Two languages, two cultures: bicultural bilinguals

Language and culture are closely intertwined and biculturalism is often associated with bilingualism (Grosjean, 2012). The language spoken by individuals and its relationship to the cultural context in which they acquire and utilize it have been subjects of inquiry. Many multilingual speakers report being different in each of their languages (Pavlenko, 2006; Dewaele, 2016), but researchers have never been able to get to the bottom of this issue and understand its real causes. The precise mechanisms underlying this phenomenon and its intricate interplay with personal experiences and social interactions continue to be a fascinating puzzle to unravel (Benet-Martínez et al., 2021) and serve as a fertile ground for exploration, holding the potential to yield deeper insights into the profound interconnection among language, culture, and individual identity.

## The fluid nature of cultural identity

Identity is a focal point in biculturalism research (e.g., Benet-Martínez et al., 2002; Benet-Martínez and Haritatos, 2005). The cultural values, traditions, and norms of both cultures significantly influence the individuals’ self-perception and sense of belonging (Berry, 2006). Balancing the expectations and practices of multiple cultures can lead to negotiating a hybrid identity that integrates aspects of both cultures while maintaining a unique selfhood (Phinney, 1990). Nevertheless, the persistent feeling of being different remains, as bicultural bilinguals are neither fully immersed in one culture nor entirely detached from the other—a state both enriching and challenging. This fluidity in cultural identity serves as a source of strength, empowering bicultural individuals to adapt and thrive in diverse environments. However, it can also evoke a sense of ambiguity and self-questioning as they navigate between cultural contexts.

Identity Negotiation Theory (INT) (Flege et al., 1999) suggests that individuals across cultures seek recognition and acceptance of their identities, influenced by cultural, social, and personal factors. Five core assumptions guide INT: (1) understanding the identity domains of communication partners boosts social self-esteem for bicultural individuals. Navigating two cultures involves addressing anxiety from emotional insecurity in culturally distant contexts (2) and (3). (4) Focuses on the importance of ingroup acceptance for trust and predictability. (5) Highlights the necessity of feeling understood, respected, and valued for successful identity negotiation. Research on acculturation and mindful identity negotiation processes (Collie et al., 2010) supports these assumptions, emphasizing the importance of affirming one’s cultural group membership while navigating bicultural identities. Bicultural individuals who effectively navigate both cultural sides and find common ground tend to experience more predictable interactions and lower anxiety levels (Gudykunst, 2005a). Ting-Toomey (2005)’s INT assumptions underscore the necessity of understanding the acknowledged identity domain of bicultural individuals and the negotiation of identity dynamics in intergroup settings. From an interactional communication perspective, bicultural individuals tend to align with perceived ingroup members when they feel secure, included, approved, and can predict interactions. Conversely, when encountering identity vulnerability, distinctiveness, and interactional discomfort, they are more inclined to distance themselves from perceived outgroup members. Dorjee et al. (2011) found that perceived identity support and positive social evaluation have a stronger association with accommodative responses than ingroup membership identification.

Communication Accommodation Theory (CAT) explores interpersonal and intergroup interactions (Giles, 1971, 1980; Giles and Coupland, 1991), driven primarily by social approval motivation. CAT distinguishes two orientations: individual, based on personal identity and social identity, based on group membership emphasis. It has evolved (Gallois et al., 2005) and been applied in various intergroup contexts (Harwood and Giles, 2005; Dorjee et al., 2011), revealing convergence and divergence strategies (Shepard et al., 2001; Gallois et al., 2005). Convergence involves matching communication strategies (Giles and Baker, 2008). In intergroup settings, bicultural individuals signal ingroup membership through language choices, like using English slang or Asian language codes. Divergence, on the other hand, employs differentiating strategies, like code-switching (Dorjee and Giles, 2005; Giles and Baker, 2008). Strauss and Cross (2005) outline specific communicative strategies, while Benet-Martínez et al. (2006) explore cultural frame-switching in bicultural identity negotiation.

Strauss and Cross (2005) examine interactional strategies employed by African Americans in interactions with mainstream European Americans to navigate co-cultural identity, complementing broader communicative strategies proposed by Harwood et al. (2005). Bicultural individuals use code-switching, buffering, bridging, and passing strategies to negotiate identity and communication in intergroup contexts. Code-switching is the adaptation of communication styles based on the cultural context (e.g., switching between English and Chinese based on the audience; Strauss and Cross, 2005). This serves as both a convergence and divergence strategy, affirming specific aspects of bicultural identity. Buffering, an identity protection strategy, involves dismissive or indifferent communication to deflect the impact of racist or ethnic jokes. Bridging uses connection strategies to engage with diverse groups, helping bicultural individuals find balance and security. Passing (Benet-Martínez et al., 2002, 2006) involves presenting oneself as a member of the dominant mainstream group. These bicultural communicative strategies provide insights into identity management in multicultural contexts, offering nuanced perspectives on identity negotiation and communication.

### The duality of belonging. The complexity of dual cultural identity

Bicultural bilinguals straddle two worlds, finding belonging in their cultures while balancing internal perspectives. They share commonalities with their communities, yet the struggle for acceptance in both cultures and a longing for authentic identity persists. Caught between these dynamics, they may face stereotypes and discrimination, making the quest for belonging emotionally challenging. Despite this, the journey fosters resilience and strengthens their sense of self. According to Grosjean (2008), becoming bicultural and fully embracing both cultures can be a challenging and lengthy process. The process of reconciling multiple cultures involves considering various factors, such as kinship, language, physical appearance, nationality, education, and attitudes (Grosjean, 2008). The outcome of this process often results in a double categorization by others, which can produce either congruent or contradictory outcomes (Grosjean, 2008). Monocultural societies tend to struggle with the notion that an individual can genuinely belong to and embrace multiple cultures simultaneously (Grosjean, 2008). The prevailing attitude often oscillates between assigning individuals to a single culture, either culture A or culture B, rather than accepting their bicultural identity (Grosjean, 2008). This limited perspective fails to acknowledge the complexity and richness of bicultural individuals’ experiences.

In order to establish their cultural identity, bicultural individuals must weigh the perceptions of both cultures and take into account personal history, identity needs, language and cultural knowledge, coping skills, and tolerance for ambiguity (Benet-Martínez and Hong, 2014). The decision-making process can lead to identifying solely with one culture, identifying with neither culture, or identifying with both cultures. These categories share similarities with Berry’s (1990) acculturation positions: assimilation, integration, separation, and marginalization.

Ideally, biculturals should accept and embrace their biculturalism as the optimal solution. However, influenced by the categorization from their cultural groups, some individuals may choose to identify solely with one culture or reject both cultures, which can lead to dissatisfaction, feelings of uprootedness, marginalization, or ambivalence. Biculturals often face negative labels such as rootless, nomadic, alienated, chameleon, and traitor, which reflect their experience of double exclusion (Grosjean, 2008, 2015). Biculturals wonder if they will ever be accepted by monocultures and be allowed to embrace their dual identity as a synthesis of both cultures while retaining their own uniqueness. Over time, many biculturals do come to terms with their biculturalism, and some may find belonging in new cultural groups (e.g., Mexican Americans or Italian Americans in North America). However, the decision-making process involved in cultural identity is complex, and unfortunately, some individuals never fully identify with both worlds they belong to (Grosjean, 2015).

The interactions and perceptions of others significantly impact the identity formation of bicultural bilinguals. Social networks, including family, peers, and communities, play a crucial role in shaping individual identities (Rumbaut, 1994). Social support and acceptance of bicultural individuals’ dual heritage and linguistic capabilities can enhance their sense of self-esteem and self-worth. However, experiences of discrimination, prejudice, or the pressure to conform to a single cultural identity may lead to identity conflicts and struggles (Houkamau et al., 2021).

## Navigating language

Language plays a significant role in the lives of bicultural bilinguals. They possess the unique ability to effortlessly switch between languages, seamlessly adapting to different social contexts [*cf*., Jylkkä et al., 2021, for a discussion about whether effort and cognitive control are required in language switching; see also Treccani and Mulatti (2015)]. However, this linguistic flexibility is not without its challenges. The feeling of “otherness” can emerge when bicultural bilinguals are caught between languages, never fully expressing themselves in one or the other. The subtle nuances and cultural references embedded within each language can be difficult to navigate, further highlighting the sense of difference that accompanies their bilingual journey.

### Language affects the way people think

According to various studies, language influences the way people think (Mykhailyuk and Pohlod, 2015). The Sapir-Whorf hypothesis (Whorf, 1956; Sapir, 1961), has contributed to our understanding of the relationship between language and thought, suggesting that language can influence a native speaker’s categorization of their experiences. Empirical research supports the concept of linguistic relativity and researchers have shared this view (Boroditsky, 2011; Ahearn, 2021). For example, when individuals switch between languages, their perspectives can change. Gender associations with nouns in different languages offer one illustration of this phenomenon. In German, the sun is feminine (die Sonne), contrasting with Spanish, where it is masculine (el sol). Similarly, the moon is masculine in German (der Mond) but feminine in Spanish (la luna). This linguistic transition influences how individuals characterize objects like bridges; German speakers use feminine adjectives for elegance, while Spanish speakers emphasize strength with masculine adjectives (Boroditsky et al., 2003).

Languages differ in expressing intentionality in accidents. English, saying “I broke my arm,” may lack clarity, while Italian, French, and Spanish prefer explicit indications like “the pencil broke” or “the pencil broke itself” (Nilsson, 2020). Spanish nuances intentionality, distinguishing unintentional events. English, e.g., “I broke the car,” lacks specificity, unlike Spanish constructions like “Rompí un coche” (I broke a car intentionally) and “Se me rompió un coche” (It happened to me that a car broke) using reflexive pseudopassive constructions (Pountain, 2003). Gibbons (2003) notes the lower intentionality expressed in Spanish’s pseudopassive construction, positioned lower on the blameworthiness scale. Additionally, Spanish has an active construction for specific intentionality lacking in English (Gibbons, 2003, p. 253).

Cultural and linguistic backgrounds also influence the attribution of blame. In Japanese culture, the concept of “amae” emphasizes dependency and interdependence, leading to a tendency to attribute blame to external circumstances rather than individuals. This differs from Western cultures, which prioritize personal responsibility, resulting in a greater inclination to assign blame to individuals themselves (Choi and Nisbett, 1998; Kitayama and Uchida, 2005).

Research suggests that the Foreign Language Effect may impact decision-making and moral judgment [for a review, see Purpuri et al. (2024)]. The foreign language might lead to reduced emotional reactions, promoting rationality and utilitarian choices (Corey and Costa, 2015). It also has the potential to decrease risk aversion and make individuals more willing to accept harm for greater outcomes (Keysar et al., 2012; Hadjichristidis et al., 2015; Winskel and Bhatt, 2020). Furthermore, it could reduce the tendency to perceive causal relationships between unrelated events and diminish common superstitious beliefs (Díaz-Lago and Matute, 2019; Hadjichristidis et al., 2019). Bilingual individuals using a foreign language might perceive dishonesty as less inappropriate and crimes described in a foreign language as less severe (Winskel and Bhatt, 2020; Alempaki et al., 2021). Recent research suggests that individuals demonstrate higher tolerance for ambiguity in their foreign language (Purpuri et al., 2023).

Emotional reactions to situations can complicate the control of intuitive processes, particularly when emotions are strong (Greene et al., 2004). Understanding the interplay between these systems and emotional responses is crucial for comprehending decision-making dynamics. Considering the context of foreign language use, it is essential to acknowledge that the learning environments for foreign languages differ significantly from those for first languages (Pavlenko, 2012). This distinction could lead to reduced emotional resonance in a person’s second language (Costa et al., 2014a, b; Iacozza et al., 2017). Such diminished emotional responses may imply a sense of emotional distance, potentially influencing judgment and decision-making processes associated with the use of foreign languages. Certainly, the attenuation of emotional responses could significantly influence how individuals perceive and act when utilizing a foreign language, warranting greater attention in discussions. Integration of research outcomes, exemplified by Caldwell-Harris and Ayçiçeği-Dinn (2021), revealing bilinguals’ inclination to align more with selfish statements and less with ethical ones in their non-native language, could offer insights into the emotional dynamics involved.

### More possible variables and specific insights

Several studies indicate that the native language elicits stronger emotional connections, images, and memories than languages acquired later in life (Pavlenko, 2005). For example, Javier et al. (1993) showed that when multilingual participants were tasked with pinpointing the most emotionally saturated language, a majority selected the one they acquired first. Additionally, bilinguals tended to offer more detailed and emotionally rich descriptions of personal memories when using the language in which the memory initially occurred. There is also a body of literature suggesting that bilinguals may perceive undesirable behaviors (e.g., lying) as easier to perform when involving a non-native language (acquired later in life) as opposed to their native one (e.g., Caldwell-Harris and Ayçiçeği-Dinn, 2009).

Therefore, the age of acquisition of the two languages mastered by bilinguals may influence how they feel when they speak either language. However, there appear to be specific aspects of the ‘feeling different’ phenomenon that derive from the characteristics of the languages used by bicultural bilingual individuals and their associated cultures, rather than from when these languages were learnt.

Dewaele (2016) examined McWhorter’s (2014) claim that bi- and multilingual individuals feel different when speaking different languages due to the different ages at which they acquired each language and the consequent differences in proficiency levels. This indeed could limit their ability to express emotions and pragmatics in the language they are less proficient in. For example, according to McWhorter, people who report feeling different when speaking their non-native language, citing differences in wit or directness, have often learned that language as adults and the reason they perceive it differently is that they have not always spoken it. However, Dewaele analyzed data from 1,005 participants and found no support for McWhorter’s assertion: the age of L2 acquisition and self-reported proficiency in L2 do not seem to be related to the extent of feelings of difference. Participants’ age, education, and anxiety in L2/L3 use were identified by Dewaele as more critical factors: in his study, these variables were all significantly and positively correlated with the intensity of the “feeling difference” experience. Participants often linked their feelings of difference to specific contexts of language use and reported these feelings to change over time, highlighting the dynamic nature of such feelings.

Overall, therefore, McWhorter’s hypothesis offers one simple lens through which to examine the “feeling different” experience. However, this simple interpretation is not supported by data. The phenomenon appears to be much more complex. Dewaele’s study prompts a broader consideration of the diverse factors that may contribute to this experience and suggests that the feeling of being different associated with the use of two different languages is not a fixed, immutable state uniquely determined by the language used (using a different language does not always result in this sensation). Instead, it is something mutable, activated by various possible triggers, among which language is just one of the possibilities (although perhaps one of the most important). Furthermore, the perceived differences do not only concern variations in wit, sharpness, or directness in expressing one’s ideas but seem to be of a deeper nature. Participants in his study reported feeling different in terms of both self-perception and behavior.

Ross et al. (2002) examined the self-perceptions of Canadian bicultural individuals when describing themselves in an open-ended questionnaire. Chinese-born participants were randomly requested to respond in either Chinese or English. As controls, Canadian-born participants, of either European or Chinese descent, responded in English. The outcomes of the language manipulation mirrored those of previous studies comparing East Asians to North Americans (e.g., Rosenberg, 1965). Participants responding in Chinese expressed more collective self-statements, lower self-esteem, and greater alignment with Chinese cultural perspectives compared to the other groups. Chinese-writing participants presented similar numbers of favorable and unfavorable self-statements in their self-descriptions, while the other groups tended to report more favorable self-statements. Chinese-writing participants indicated comparable levels of positive and negative mood, whereas the remaining groups reported higher positive mood.

Ervin’s (1964) study on Japanese-American bilingual women found language-dependent variations in sentence completion. When tasked with completing sentences in both Japanese and English, participants provided markedly distinct endings based on the language employed. For instance, when prompted with the sentence “When my wishes conflict with my family,” responses in Japanese indicated a perception of “it is a time of great unhappiness,” whereas responses in English reflected a sentiment of “I do what I want.”

Ringberg et al. (2010) conducted a study involving a cohort of Hispanic-American women, all proficient in both languages, but varying in their levels of cultural identification. The researchers observed shifts in participants’ self-perception depending on whether they were interacting with members of their native culture (and utilizing their native language) or they were acting within an environment dominated by a different culture (and using another language). Additionally, when participants were tasked with interpreting advertisements featuring women, their perceptions differed based on the language employed: women in Spanish-addressed ads were viewed as more self-sufficient and extroverted, while those in English-addressed ads were perceived as more traditional, reliant on others, and family-oriented. Notably, this language-triggered “frame switching” appeared to occur involuntarily and was observed solely among biculturals, rather than monocultural bilinguals.

Caldwell-Harris et al. (2011) showed that Chinese—English bilinguals residing in the US tended to perceive emotional expressions in their native language, Mandarin, as stronger compared to expressions in their second language, English. Despite this perception, they preferred to express their emotions (e.g., saying “I love you”) in English due to perceived social constraints being more relaxed in English-speaking environments. Electrodermal monitoring conducted on a similar sample (Caldwell-Harris and Ayçiçeği-Dinn, 2021) revealed that bilinguals with proficient abilities in both Mandarin and English exhibited similar physiological reactivity (skin conductance responses) to emotional expressions in both languages, except for endearments where English expressions elicited larger responses. This difference could be attributed to cultural norms, as English-speaking societies encourage more open expression of positive emotions compared to Asian Cultures.

All these findings suggest the feeling of being different associated with the language used results from the differential activation of values, expectations and aspirations, rather than simply to a lesser or greater ability to express them in the two languages, due to acquiring these languages at different ages and times. In bicultural individuals, different (or partially non-overlapping) cultural-specific knowledge appears to mediate the distinct experiences corresponding to different cultural identities (e.g., Eastern and Western; Ross et al., 2002). In our view, these partially non-overlapping structures may allow culturally-specific memories and response patterns to be more activated when using a given language compared to when using another.

Dewaele (2016) reported the answers of different bilingual bicultural individuals to a questionnaire in which they were asked to describe their feelings of being different when they speak different languages. He indeed points out that, although participants in his study did not always fully understand the reasons behind their feeling different experience, many of them seemed to be aware that this experience is somehow related to the different cultural values and habits linked to their languages. For instance, Angelika, a 24-year-old female with Swedish as her first language, English as her second language, Japanese as her third language, and French as her fourth language, articulated her experience by stating that, when speaking in Japanese, she adapts to the Japanese culture extensively. Her voice elevates, adopting a more feminine tone, reminiscent of Japanese women (“I speak with a light voice just like a Japanese woman”). In contrast, when conversing in Swedish or English, her demeanor is notably more direct.

Angelika explicitly contrasts her feelings when speaking an Eastern language compared to when speaking a European language (she does not feel different when speaking Swedish vs. English, but when speaking Japanese compared to when she speaks one of her European languages). In fact, the majority of studies on the feeling of difference have focused on the experience that bicultural bilinguals have when speaking two languages associated with very different cultures, such as those of a Western and an Eastern culture. This brings us to an interesting question. How much does similarity versus difference between the spoken languages and associated cultures influence the experience of feeling different? When two languages belong to broadly similar cultures, this might reduce the difference between the cultural values, behavioral patterns and expectations being primed by the used language. When the two languages belong to very different cultures, then the perceived difference might increase. To the best of our knowledge, however, no study has yet explicitly investigated the impact of similarity between the cultures of bicultural bilingual individuals on the nature and intensity of the feeling different experience when they speak the languages associated with these cultures.

## Discussion

The complex and multifaceted phenomenon of “feeling different” among bicultural bilinguals stems from the interconnection between cultural and linguistic identities. Biculturalism, integrating two distinct cultures, and bilingualism, mastering two languages, shape their experiences, offering opportunities for adaptation and growth but also presenting challenges.

The language people speak can mold their thoughts, perspectives, and decision-making processes, thus having a significant impact on the experience of individuals who have come into contact with more than one culture and use different languages associated with these cultures in their daily lives (Mykhailyuk and Pohlod, 2015).

The negotiation of cultural identity is central to the experience of these individuals, requiring constant adaptation and self-reflection. They navigate diverse belief systems, values, and traditions, forging a hybrid identity while dealing with moments of exclusion and conflicts between societal expectations and personal values. Our analysis suggests that the “feeling different” experience can be indeed perceived both negatively and positively. While it may entail feelings of exclusion or marginalization within one’s own community, it also often leads to positive outcomes, enriching personal development and contributing to societal diversity. When not experienced with discomfort, this feeling of being different linked (even if not exclusively) to the use of different languages can lead to a sense of pride, fulfillment and gratification for one’s dual (yet simultaneously unique) identity.

Investigating how stereotypes, experiences of discrimination, or marginalization, but also positive feelings resulting from the acknowledgment of belonging to two different worlds, impact identity negotiation and bicultural identity integration among bicultural individuals is crucial. The well-being of bicultural bilinguals is significantly influenced by self (internal) and others’ (external) perceptions and acceptance. Social support and recognition of their dual heritage enhance self-esteem and these positive feelings, while discrimination or pressure to conform can lead to identity conflicts. Accordingly, by continuing to investigate biculturalism-related phenomena, we can enhance our understanding of bicultural experiences and work toward fostering inclusive environments that honor and celebrate diversity.

Further studies could explore the role of familial and societal support systems in fostering bicultural identity development. Understanding how these factors influence the negotiation of multiple cultural identities can inform interventions and support mechanisms for bicultural individuals.

While acknowledging the profound impact of biculturalism and bilingualism on the lived experiences of bicultural bilinguals, this article emphasizes the need to better understand the intricate dynamics of their identity negotiation and cultural adaptation. By synthesizing insights from various disciplines such as sociology, linguistics, and psychology, we aimed to offer a holistic perspective that explores the intersectionality of factors shaping the sense of difference among bicultural bilinguals.

Embracing biculturalism in our highly interconnected world can foster understanding and appreciation for diverse cultural outlooks, contributing to a more inclusive society. The feeling of being different depending on the linguistic context one is immersed in is an interesting phenomenon, but it is much more than mere curiosity. It offers valuable insights into the actual reality of bicultural bilinguals, shedding light on the complexities of their cultural identity formation. By studying this phenomenon, we gain a deeper understanding of the intricacies of the bicultural bilinguals’ experience, allowing us to better comprehend their unique perspectives and challenges. Further research into this phenomenon can be useful to uncover new insights into the multifaceted dimensions of this experience.

## Data availability statement

The original contributions presented in the study are included in the article/supplementary material, further inquiries can be directed to the corresponding author.

## Author contributions

SP: Writing – original draft. CM: Writing – original draft. RF: Writing – original draft. BT: Writing – original draft.
